# “In the Sport I Am Here”: Therapeutic Processes and Health Effects of Sport and Exercise on PTSD

**DOI:** 10.1177/1049732317744533

**Published:** 2017-12-03

**Authors:** Clemens Ley, María Rato Barrio, Andreas Koch

**Affiliations:** 1 Universität Wien, Wien, Austria; 2 Asociación para la Cooperación, la Convivencia y la Investigación (ACCI), Madrid, Spain

**Keywords:** exercise / physical activity, refugee, mental health, trauma, depression, case study, attentional focus, motivation, distraction, exposure, qualitative, Austria

## Abstract

Current evidence suggests positive effects of exercise on posttraumatic stress symptoms; however, knowledge about how these effects are achieved is limited. Thus, this study aims to contribute to a more holistic understanding of these effects. We performed a single case study of a war and torture survivor, who was diagnosed with posttraumatic stress disorder (PTSD) and depression, and who was participant of the sport and exercise therapy program *Movi Kune*. Participant observation was conducted as well as semi-structured interviews with the participant and his psychotherapist. Data analysis resulted in the proposal of different processes: The focus on bodily sensations related to an exposure effect, contributing to improvements in body awareness, coping behavior, and affect regulation, whereas the focus on playing related to an improved performance, presence, enjoyment, and mastery experiences, pointing toward distraction and motivational-restorative effects. The findings also advice to be cautious as participants may be exposed to negative sensations and trauma-related triggers.

In conflict regions and humanitarian crises, people are subjected to experience unimaginable atrocities and repeated violations of their basic human rights (e.g., persecution, violence, abuse, torture, and humiliation; Hebebrand et al., 2016; United Nations High Commissioner for Refugees [UNHCR], 2015). People seek protection primarily in their home countries or in neighboring countries; however, many refugees are forced to flee further to other states and experience further violations of their basic human rights (UNHCR, 2015). Given these extreme experiences, refugees have often suffered in their home countries and during forced migration, host countries are encouraged to provide protection as well as stimulate health and social inclusion (Hebebrand et al., 2016). In this context, the current study aimed to examine how sport and exercise therapy can facilitate meaningful health-related processes for refugees who have survived war and torture.

## Health of War and Torture Survivors

The health of refugees from conflict regions is heavily affected by their past and current experiences and situation. Premigration traumatic events (e.g., violence, torture, combat experiences), as well as forced migration and postmigration stressors (e.g., resettlement difficulties, immigration detention, temporary protection, and acculturative hassles), contribute to severe psychological distress and loss of resources (e.g., loss of social network; limited access to health care, educational, and occupational opportunities; language barriers; unfamiliar environment; Drožđek, 2015). This imbalance between stressors and available resources often results in major health problems, that is, a high prevalence of posttraumatic stress (PTSD), depression, and anxiety disorders. For example, Kartal and Kiropoulos (2016) concluded that acculturative stress relating to postmigratory experiences was associated with increased PTSD and anxiety symptoms among refugees living in Austria. People living with PTSD often experience fatigue, lack of motivation, and negative mood states, as well as psychosomatic problems, for example, severe pain sensations and somatization symptoms (Drožđek, 2015; Hebebrand et al., 2016; Van der Kolk, 2014). In daily life, traumatic experiences are often present in their thoughts in the form of intrusive memories from the past or unwanted flashbacks, reexperiencing the trauma; as a consequence, they face problems with sleeping, concentration, and cognitive performance, and are often less present in the *here and now* (Maier & Straub, 2011; Van der Kolk, 2014).

As a result of PTSD, people may show behaviors like social withdrawal or isolation to avoid fearful sensations and situations that may trigger trauma-related memories, anxiety, or distress. Such avoidance or self-protection behavior may reduce inclusion and social interaction with others. In turn, lack of inclusion may negatively affect their health state, for example, feeling lonely and excluded from social interaction (Lincoln, Lazarevic, White, & Ellis, 2015). In this regard, the sociopolitical discourses concerning refugees may also negatively affect their health. For example, the sociopolitical climate in Austria during the so-called “refugee crises” in 2015 and 2016 was polarized, including, on one hand, discourses about welcoming the recently arrived refugees and support from individual citizens and nongovernmental organizations, on the other hand, discourses about the need to close borders and migration routes and perform an extended and accelerated deportation (Rheindorf & Wodak, 2017). Discourses such as these fuel the fear of deportation, anxiety, and a sense of insecurity, and may negatively influence the psychosocial health of refugees.

Although trauma is often thought of as a psychological event, the physical bodies of war and torture survivors are often affected in multiple ways: having been subjected to direct physical aggression during traumatic events and remembering psychological traumata as well (Rothschild, 2000). Notably, hyperarousal, chronic muscle contraction, back and neck hardening, chronic pain, and unnatural body postures are frequent symptoms associated with the—often dissociated and disconnected—bodies of war and torture survivors (Rothschild, 2000; Van der Kolk, 2014). Without a healthy embodiment and body awareness, the possibility of experiencing a sense of security and pleasure in the body and the capacity to feel grounded and centered is often impaired (Rothschild, 2000).

Thus, any psychosocial support of and therapy with those affected by trauma should consider the psychological, the social, and the physical. In this regard, sport and exercise may prove to yield beneficial effects to war and torture survivors. To provide a rationale for study, the current literature examining sport and exercise with war and torture survivors and people living with PTSD will be reviewed below.

## Sport and Exercise With War and Torture Survivors and People Living With PTSD

The scientific literature on physical activity and body-related work with traumatized refugees, torture and war survivors is scarce and at the same time diverse, coming from a wide range of disciplines and therapeutic orientations, including sport and exercise therapy; yoga, qigong, and t’ai chi; dance movement therapy (DMT), psychomotor therapy, play therapy, creative and art therapies; integrative and concentrative movement psychotherapy; and other body- and movement-orientated psychotherapeutic approaches (cf. Levine & Land, 2016; Ley & Rato Barrio, in press). More research has been conducted on the effects of physical exercise on a wider range of individuals diagnosed with PTSD, including combat veterans and individuals suffering from other traumatic events, for example, the sudden death of a person close to them, a severe accident, or the diagnosis of an illness or disability. For example, a recent quantitative meta-analysis, which included four randomized-controlled trails (RCT), reported a small to moderate effect size of exercise on reducing PTSD and depression symptoms in people with PTSD (Rosenbaum et al., 2015), suggesting the inclusion of aerobic and resistance training as well as yoga-based exercises as adjunctive treatments. Caddick and Smith (2014) highlighted in a systematic review of quantitative and qualitative studies the various outcomes of sport and physical activity on the well-being of combat veterans in the aftermath of physical and/or psychological combat trauma and PTSD, including active coping and *doing things again* (after inactivity), a focus shift on abilities, positive affective experience, improved quality of life, increased determination and inner strength, a sense of achievement or accomplishment, social well-being, and a source of *motivation for living*. Similarly, Levine and Land (2016) presented a meta-synthesis of qualitative findings, including nine studies on DMT for individuals with trauma. In contrast to sport and exercise therapy, DMT uses the body and movement within a psychotherapeutic approach. Four crucial themes were concluded:

(a) creating awareness of the mind-body connection; (b) increasing the range of movement (for the purpose of efficacy, empowerment, and reclaiming the body); (c) creating a new and healthy relationship with the self, therapist, or group through the movement process; and (d) creating a new and healthy relationship with movement. (Levine & Land, 2016, p. 14)

These two reviews showed the need for further qualitative studies to gain a more profound and holistic understanding of the processes and effects of sport and exercise.

Van der Kolk et al. (2014), from a 10-week RCT with 64 women suffering from chronic, treatment-resistant PTSD, concluded that “yoga significantly reduced PTSD symptomatology, with effect sizes comparable to well-researched psychotherapeutic and psychopharmacologic approaches” (p. e559). Trauma-sensitive yoga is associated with processes of body awareness and mindfulness, which might be beneficial in dealing with hyperarousal and increasing attention, emotional awareness, and affect tolerance (Van der Kolk et al., 2014). However, Fetzner and Asmundson (2015) critically discussed the potential use of exercise in changing the attentional focus toward (e.g., through body awareness, mindfulness) or away from somatic arousals and bodily sensations (e.g., through distraction). The authors not only concluded that both effects might be possible, but also that other processes such as experiencing “a sense of accomplishment,” a “brief reprieve from everyday anxiety,” and “behavioural activation” must be investigated in the study of physical exercise with people living with PTSD (Fetzner & Asmundson, 2015, p. 310), indicating that one specific process or mechanism cannot explain the psychological effects of sport and exercise (cf. Faulkner, Hefferon, & Mutrie, 2015).

Although sport and exercise do appear to show some promising effects in trauma survivors, refugees, war and torture survivors often disengage from sport and exercise as a consequence of traumatic events as well as of migration, resettlement, and acculturation challenges (O’Driscoll, Banting, Borkoles, Eime, & Polman, 2013). Important correlates for physical activity in culturally and linguistically diverse migrant populations include their attitudes toward physical activity, self-efficacy beliefs, and knowledge; general level of motivation, fatigue, and affective state; feelings of isolation and lack of social support; socioeconomic challenges, structural barriers, sociocultural norms and practices as well as unfamiliarity with the environment (O’Driscoll et al., 2013). However, participation is also affected by mental health and symptoms of PTSD, depression, and anxiety (Vancampfort, Stubbs, Venigalla, & Probst, 2015). For example, sleep disturbance is common among war and torture survivors; low sleep quality is associated with lower physical activity in people with PTSD (Talbot, Neylan, Metzler, & Cohen, 2014). Furthermore, exercise might produce bodily sensations, such as increased heart rate, muscle tension or pain, sweating or shortness of breath that are also associated with previous traumatic events or distress. In that regard, dropping out or physical inactivity can be seen as avoidance behavior, that is, to avoid feared bodily sensations (Levine & Land, 2016). Assis et al. (2008) argued that “subjects diagnosed with PTSD stop participating in society and no longer take part in activities they used to enjoy; this inaction may lead to depression and social isolation” (p. 476). The authors found PTSD patients preferred to choose individual sports over group activities like soccer, handball, and dancing after the onset of PTSD even if they had been involved in team sports before. Such choices might be affected by the consequences of PTSD, fear, anxiety, attachment disorder, or feelings of isolation (Assis et al., 2008). Hence, social interaction could have important positive benefits for this population (Assis et al., 2008; Caddick, Smith, & Phoenix, 2015; Carless, Sparkes, Douglas, & Cooke, 2014).

## Purpose of the Study

Although the potential benefits of sport and exercise seem promising, little is known about how these benefits may be gained in people with mental health illness (Faulkner et al., 2015), particularly in people living with PTSD. There is a need to understand holistically and in-depth how sport and exercise should be facilitated to meet individual and collective needs, which precautions are to be considered, how potential benefits toward recovery can be gained, and which processes relate to which effects (cf. Caddick & Smith, 2014; Day & Wadey, 2016; Fetzner & Asmundson, 2015; Levine & Land, 2016). Understanding these issues would increase the possibility of effectively and adequately planning and implementing sport and exercise programs in this population.

In response to this paucity of knowledge, this study aims to describe and explain therapeutic processes and effects taking place in a sport and exercise program with war and torture survivors. War and torture survivors face serious health issues with a high prevalence of PTSD, depression, and anxiety disorders due to their experiencing traumatic events, forced migration, and acculturation challenges in a new, culturally and linguistically different society. Therefore, we conducted a holistic, single case study attempting to provide an in-depth and nuanced account of a single participant, and construct a more holistic understanding of the complex phenomena of diverse processes and effects of sport and exercise in relation to specific individual needs and recovery processes (cf. Day & Wadey, 2016; Stake, 1995; Yin, 2014).

## Method

The study was part of a broader research project which was approved by the university ethics committee (No. 00049). We conducted a case study as defined by Creswell, Hanson, Clark Plano, and Morales (2007), exploring “a bounded system (a case) . . . over time through detailed, in-depth data collection involving multiple sources of information (e.g., observations, interviews, . . . ) and report[ing] a case description and case-based themes” (p. 245). A constructivist and interpretivist perspective framed this case study recognizing that knowledge is socially constructed and subjective, and that multifaceted realities exist (Sparkes & Smith, 2014). As in the case study methodology of Stake (1995), which aligns to a constructivist and interpretivist orientation (cf. Harrison, Birks, Franklin, & Mills, 2017; Lauckner, Paterson, & Krupa, 2012), the meaning and understanding of the experiences in context were central as well as the researchers’ interpretive and interactive roles in the research process. A constructivist perspective on the present case study commended the holistic exploration of the case to be based on the descriptions and interpretations of the participant, the observers, and the therapist, using multiple sources of information, and the active engagement of the researchers with the subject and context of the case study (cf. Sparkes & Smith, 2014; Stake, 1995).

The bounded system of this case study comprised the *subject* (or practical unit), that is, the person, the place, the program, and so on, as well as the *object*, that is, the analytical frame (Thomas, 2016). The analytical frame developed from the interest on the complex phenomena of processes and effects of sport and exercise on people with mental health illness, particularly PTSD. The subject of this study was a war and torture survivor (diagnosed with PTSD and depression), participant of the sport and exercise program *Movi Kune*, which was delimited by time, place, and activity. Due to the heterogeneity of the participants with regard to their individual context, that is, their social-cultural background, current living and working situation, lived traumatic experiences, physical condition, health and well-being as well as recovery process, we felt it necessary to conduct a holistic, single case study (Yin, 2014; cf. Day & Wadey, 2016). A holistic, single case study allows a more in-depth and nuanced account of a single participant and a more holistic understanding of the complex phenomena of the perceived processes and effects of sport and exercise in relation to the specific individual situation, for example, needs, motivation, health, and recovery processes (cf. Day & Wadey, 2016; Eisenhardt & Graebner, 2007; Yin, 2014). Although the case itself was of interest, the case study was predominantly instrumental (cf. Stake, 1995), aiming to provide insides on the issue in question and contribute to the explanation of processes and effects in sport and exercise therapy with war and torture survivors and people living with PTSD.

### Participant

The case study explored the experiences of a single participant of the sport and exercise therapy program *Movi Kune*. The study participant was selected out of the total of 14 men who participated at least once in the male group in 2014 (on average six participants were present per session). All participants were more than 18 years old and gave voluntary informed consent to participate in the research. The existence of thick descriptions and rich data relating to the person was crucial in the selection of this participant for this study to learn the most from the case (cf. Stake, 1995). From a constructivist stance, it was important that (a) the observers recorded enough data regarding the participant; (b) the participant was available for a final interview, reflecting on his subjective experiences during the program; and (c) the participant’s therapist was available for an in-depth interview. The inclusion of these three different perspectives allowed furthermore for convergence and triangulation of data from multiple sources, which is an important aspect of case study methodology (Stake, 1995; Yin, 2014).

### Intervention: Sport and Exercise Therapy Program Movi Kune

Since 2013, the sport and exercise psychology department (University of Vienna, Austria) and the care center for torture and war survivors Hemayat have been working together in this project, offering sport and exercise therapy as an adjunctive offer to psychotherapy. Each year, war and torture survivors in the care of Hemayat participated in gender-separated groups (on average 5–10 participants per group). The male group of 2014 was facilitated by two trainers/facilitators and one sport and exercise therapist, and was accompanied by a trauma-expert. The intervention was conducted for 3 months, with two weekly sessions of 90 minutes each. The contents and strategies were multimodal in nature and were documented up-front and revised after each session, as the program was continuously adapted to the needs and interests of the participants to augment motivation and adherence to physical activity. For example, in the intervention group of this study, the participants often expressed their wish to play basketball, although different sports and games (Tchoukball, Ultimate Frisbee, and other ball games) were introduced. The program was based on sport, exercise, and movement therapy principles, including the dimensions of *training, learning*, and *experiencing*, a perspective on Salutogenesis and health literacy (see Ley, Lintl, & Movi Kune Team, 2014). Various tools were applied, including modified sports, dance, and games; respiration and relaxation techniques; movement tasks, body awareness and grounding exercises; and endurance, resistance, coordination, and mobilization exercises. Nonverbal methods were combined with verbal techniques, applying mainly person-centered communication. Group processes were managed by regulating the degree and method of social interaction and physical contact, by providing the individual choice to opt-out and opt-in, and by fostering self-regulation of the training load, own level of engagement, and role in the team. The continuous participation of a trauma-expert in the intervention was crucial to work within the limits of competences and to deal in the best possible way with potential trauma triggers and exposure to negative experiences (Ley, Krammer, Lippert, & Rato Barrio, 2017; Ley & Rato Barrio, 2017).

### Data Production

“Case study research builds an in-depth, contextual understanding of the case, relying on multiple data sources rather than on individual stories as in narrative research” (Creswell et al., 2007, p. 245). Aligned with Stake’s (1995) approach, we used interviews and observation as data production methods. Semi-structured interviews of approximately 30 minutes were conducted with the participant at the beginning and end of the intervention phase. The initial interview took place at Hemayat, as this was a well-known place for the participant. The final interview took place at the University, as that was the place where the intervention was conducted. Choosing places that were familiar and trustworthy to the participant was important to avoid stressful situations and provide a feeling of safety, thus allowing a deliberate expression of subjective perceptions and experiences. Both interviews were led by the principal researcher (Clemens Ley), who is experienced in conducting interviews with individuals with various health problems and social-cultural backgrounds (e.g., survivors of violence in Guatemala, people living with HIV in South Africa). The principal researcher conducted the interviews together with the trauma-expert and an interpreter from Hemayat, both of whom are experienced in working with war and torture survivors. The initial interview explored the participant’s expectations, previous experiences, attitudes toward sport and exercise, perceived barriers, and health issues, as well as with ethical issues (e.g., the risks and benefits of participation, the rights of the participant, and procedures for the safeguarding of data and the protection of the participant). The final interview included questions about perceived changes, subjective meaningful processes, key events, positive and negative experiences, barriers, and his recommendations for future programs. A semi-structured interview guide was used in a flexible way to stimulate reflection and more detailed descriptions, as well as to provide the participant the freedom to express himself about subjective meaningful experiences. He was also asked to give concrete examples from the sport and exercise sessions and to reflect about possible connections between perceived changes, responsible processes or happenings, and contextual aspects. The interviews were not recorded to avoid distress and anxiety. Instead, quick notes were taken during the interview to not only ensure capturing the full range of aspects and arguments but also not to interrupt the conversation process, allowing the participant to develop his story through an active listener (cf. Carless et al., 2014). Directly after the interview, the notes were completed, including as much as possible the recollection of direct first-person quotes from the participant. In addition, the interviewer included in the notes own observations (e.g., body language) and possible interpretations which he discussed *in situ* with the participating therapist, debriefing, scrutinizing, and contrasting perceptions, aiming to understand and grasp the meaning of what the participant said or attempted to express (cf. Day & Wadey, 2016; Hill et al., 2005).

In addition to the participant interviews, a 1-hour semi-structured interview was conducted with the participant’s psychotherapist, who was also the participating trauma-expert in his case. This interview was audio-recorded and transcribed verbatim. The following topics were discussed: the contextual background and health conditions of the participant, processes of and perceived changes resulting from the sport and exercise program, their therapeutic meaning, potential relations between processes and effects, possible influencing factors (e.g., medication, psychiatric interventions, and living conditions), synergistic effects with psychotherapy, and overall health and inclusion outcomes (outside of the program).

Participant observation was conducted in each session by two trainers, one sport and exercise therapist and one trauma-expert (psychotherapist). The two trainers and the sport and exercise therapist participated actively in the activities and interchanged the facilitating roles throughout the session (e.g., one was responsible for warm-up and strength exercises, another for the sport game, and the third for the relaxation exercise); the trauma-expert also participated actively in the sports and exercises, being always available for an individual chat and counseling. In this way, all observers formed trustworthy relationships with the participant and had the opportunity for in-depth observation (cf. Jones, Brown, & Holloway, 2013). Observation was implemented predominantly in an explorative and open way, without using any structured category list at the moment of observation. The observations were written down in the form of field notes after each session, as the writing of notes during the sport and exercise session was perceived as inappropriate (cf. Thorpe & Olive, 2016).

Importantly, the field notes contained descriptions and reflections about nonverbal and verbal expressions of emotions, experiences, behavior, happenings, and events, also including notes about verbal communication during the sport and exercise session, for example, asking about current thoughts, perceptions, expectations, or feelings, and spontaneous chats while running together. The information was always contextualized, including the moment of occurrence, activity, interaction with other participants and trainers, or other influencing factors (e.g., place or weather).

### Data Analysis

Data analysis aimed to search for meaning and understanding of the case, to provide thick descriptions, and to tell a story about the case which is holistic and in-depth and which allows a naturalistic generalization by the user (Lauckner et al., 2012; Stake, 1995). Furthermore, a bottom-up approach was adopted to build and abstract theoretical concepts from the data, aiming to explain therapeutic processes and health effects of sport and exercise. In the inductive analysis of the data, we used analytical techniques such as open and focused coding, searching for patterns, thematic grouping of findings, and reflective and contextualizing memo-writing as well as comparison and triangulation techniques to build the theoretical concepts (Lauckner et al., 2012; Stake, 1995). The story and the constructed concepts were then compared with existing theories and concepts.

**Figure 1.**
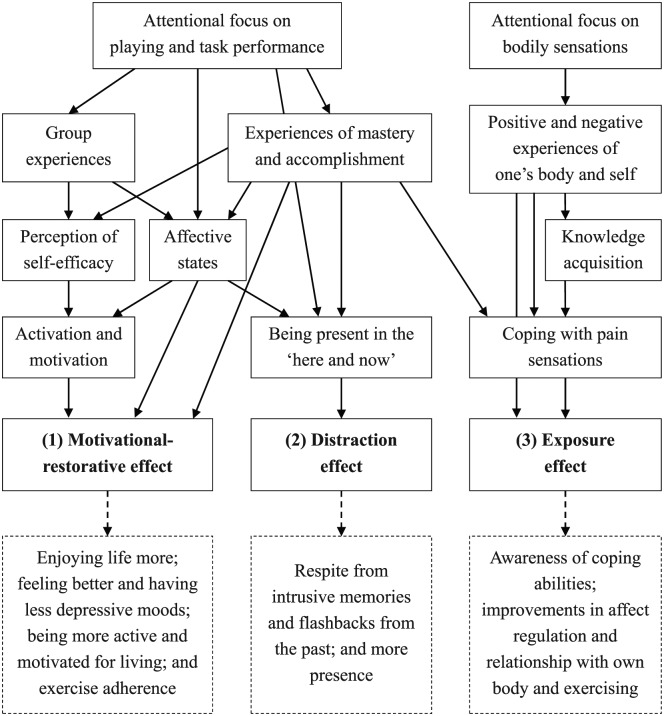
Conceptual map of themes.

All data were written in German and analyzed using the software program *Atlas.ti* (v7) by two researchers (Clemens Ley and Andreas Koch) who had engaged intensively with the subject of the case study in the program. The third researcher (María Rato Barrio) audited and stimulated critical reflection in the data analysis process from an outsider point of view (see below *Reflexivity*). Once familiar with the data, inductive data analysis started with open coding and searching for patterns. With increased saturation, the assigned contents of each code were revisited, and, if necessary, codes were redefined and reworked. Focused coding was then applied, increasing saturation of data in the emerged codes. Furthermore, codes were compared and linked between each other, searching for further patterns and grouping findings, developing themes and concepts (cf. Jones et al., 2013; Lauckner et al., 2012; Stake, 1995). In this process, we used reflective and contextualizing memo-writing and mapping of themes as well as constant comparisons of the codes, themes, concepts, and literature, iteratively questioning the findings (cf. Jones et al., 2013; Lauckner et al., 2012). We built the theoretical concepts through analyzing and mapping of relationships between the themes (see *Results of data analysis*) attempting to explain how and why the effects happened, identifying potential processes and effects (see Figure 1). Furthermore, literature was reviewed and discussed processual (Jones et al., 2013). Triangulation of the multiple sources of information and researchers helped not only to scrutinize the trustworthiness of the interpretations, but also to question meaning and understanding by identifying different perspectives (cf. Stake, 1995). Different perspectives were discussed in the reflexive and consensus processes among the researchers and with the other trainers and trauma-experts as well. All these persons added valuable opinions from their respective perspectives, scrutinized assumptions and interpretations as well as emerging themes and concepts, and together corroborated the findings, thus contributing to the construction of the case.

### Reflexivity and Generalizability

As is common in qualitative research, we reflected upon our potential biases about this research. We acknowledged that we all believed in the benefits of the *Movi Kune* program and the intervention methodology, with all its strengths, difficulties, and shortcomings, and that we were eager to improve it through critical analysis and discussion. Furthermore, we admitted that our sociocultural background differed from the background of the subject of the case study, which might limit our understanding of the experiences and meaning. The participant was from a South Asian country and grew up in a rural area. The researchers of the study were all mainly socialized and culturalized in Western societies in Europe, though we probably gained some degree of sociocultural sensitivity through working with diverse population groups. Two of us (María Rato Barrio and Clemens Ley) had extensive work experience in culturally and linguistically diverse populations and postconflict countries, including many years of living abroad, and backgrounds in anthropology and cross-cultural psychosocial intervention in complex emergencies. Our previous experiences (e.g., in dealing with feelings of strangeness in intercultural encounters, in attempting to grasp local knowledge and sociocultural meaning and understanding, in ethic-emic perspectives and reflexive work) helped us in constantly questioning our preconceptions, our subjective and socioculturally marked perceptions, and potential influences on data production and analysis processes.

We wrote reflexive field notes throughout the research process and reflective memos during data analysis using *Atlas.ti* (cf. Harrison et al., 2017). We attempted to triangulate own perceptions and interpretations by asking other trauma-experts, therapists, and participants, for example, in interviews or informal talks during or after the sport and exercise sessions, about their views on the issue in question or to comment on our perceptions and interpretations. Two of us (Clemens Ley and Andreas Koch) were deeply involved in the implementation of the program and intensively engaged with the subject of the case study during data production and program delivery, developing a profound and trusted relationship with the participant. Playing and exercising together helped to foster an interaction among *equals*—performing the same tasks, following the same rules, playing together in a team, and sharing experiences and emotions—thus avoiding barriers of a hierarchical therapist–patient or researcher–participant relationship nature (e.g., unequal power-relationship, unidirectional relationship). This intersubjective relationship induced an increased openness, forthrightness, and intimate talks. For example, the participant described the participating trauma-expert as his “friend,” though the relationship was clearly delimited to the sport and exercise program and support at the care center Hemayat. In contrast, the third researcher (María Rato Barrio) did not participate in the program delivery and did not know the participant other than through the data. Her role was more like a critical outsider (ethic perspective), an “auditor to check the work of the primary team of judges and minimize the effects of groupthink in the primary team” (Hill et al., 2005, p. 197), stimulating critical reflections among the researchers. Hence, several persons were involved in producing and interpreting the data and in discussing meaning and understanding from multiple perspectives. We used consensus processes by critically discussing individual perceptions, preconceptions, disagreements, and diverse perspectives, aligning to the consensual qualitative research approach of Hill et al. (2005). Besides the research team, we also included the other trainers and trauma-experts of the program in these reflexive and consensual processes, for example, discussing the meaning and understanding of the participants’ verbal and nonverbal expressions with the participating therapist shortly after jointly conducting the final interview. Accordingly, Hill et al. (2005) argued that the

variety of viewpoints and experiences among the team members may help unravel the complexities and ambiguities of the data. Thus, a common understanding of the data is sought while preserving the right of individual team members to hold differing worldviews. (p. 198)

We consider that this instrumental case study allows for analytical theorizing (Day & Wadey, 2016) or analytic generalization (Yin, 2014) from the data to theory—as opposed to a statistical generalization to a population—through using analytical strategies, building of theoretical concepts on the relationships of processes and effects, and drawing comparison with existing theories and concepts (see “Data Analysis”). Furthermore, we suggest the transferability of the findings to similar cases (cf. Day & Wadey, 2016; Sparkes & Smith, 2014) and align with Stake’s concept of naturalistic generalization, which proposes that the user (i.e., reader) naturally compares and transfers the case with own experiences, knowledge, and cases (Stake, 1995). Thus, we attempted to provide a thick description of the case in context, facilitating for the user the basis for naturalistic generalization and own judgment of transferability (cf. Day & Wadey, 2016; Stake, 1995).

## Findings

The interview with the participant was limited in length and depth, due to major challenges on the part of the participant to concentrate, recall, and express verbally his perceptions and experiences. These challenges were strongly influenced by his personal situation and health problems. However, current verbal and nonverbal expressions (e.g., comments, gestures, facial reactions) from the participant during the sport and exercise sessions were captured frequently in the observers’ field notes, providing an additional account of the participant’s experiences. Participant observation provided thick descriptions of social interactions, task performance, skills, levels of activation and participation, attentional focus, and affective response. Data from the interview with the therapist were rich in critical reflections and contextualized with individual background information.

### Case Description

Rashid (pseudonym) is a young man, around 20 years old, who was born as an eldest son in a rural area of a conflict-torn South Asian country. His family lived in very poor conditions. Rashid had to witness the murdering of his parents. He and his brother survived, but were separated. After having searched for half a year without success, he assumed the death of his younger brother and decided to flee his country. He was able to reach Greece where he was imprisoned. Later, he lived homeless with other refugees in Athens. From there, he was brought through Macedonia, Bulgaria, and Hungary to the Austrian initial reception center in Traiskirchen. At this time, he was still underage. He was diagnosed with PTSD according to the International Classification of Diseases, tenth edition (ICD-10) F43.1, which was still persistent at the time of participation in the program. In addition, he was diagnosed with a recurrent depression disorder according to ICD-10 F33.2 and as an acute suicide risk. He was taking various neuroleptics and antidepressant medication.

Rashid joined the sport and exercise program in 2014 as an adjunctive therapy, advised to do so by his psychotherapist. At this time, he was not practicing any sport or exercise. He took part in the entire program (3 months) with a certain regularity, although he had several absences due to illness, psychiatric interventions after suicide attempts, and problems with the accompanied transport, thus resulting in a total adherence rate of 41% and an adherence pattern similar to many other participants.

### Processes and Effects of Sport and Exercise

Analyzing the emerging themes and relationships between the themes, we constructed three core concepts theorizing different processes in relation to potential effects of the sport and exercise program, that is, (a) a motivational–restorative, (b) a distraction, and (c) an exposure effect. Figure 1 shows the conceptual mapping of the themes, visualizing potential relationships among the processes and effects through the arrows.

Data analysis showed, for example, that narratives about Rashid’s positive *affective states* were often linked to *mastery experiences and accomplishments*, positive *group experiences* (e.g., feedback from others, a sense of belonging), and an *attentional focus on playing*. Pain sensations were reported recurrently, particularly relating to an *attentional focus on bodily sensations* during physical exercises. Furthermore, two different attentional foci were identified which seemed to occur in different activities (playing a ball game vs. exercise training) and which seemed associated with different processes and effects (see Figure 1). The themes and relationships are discussed in more detail below.

#### Motivational-restorative effect

The findings suggest that the following themes, that is, mastery experiences, positive affective states, group experiences, and activation, relate to a motivational and restorative effect on Rashid, contributing to an improved sense of well-being, that is, enjoying his life more, having less depressive moods, being more active and motivated for living, and toward his exercise adherence. Furthermore, the attentional focus on playing and present task performance seemed crucial to these themes, as we describe below.

##### Affective states

In the first half of the program, Rashid showed little positive emotion. He was perceived by observers as tired, anxious, withdrawn, reticent, lethargic, and with a prevailing depressive mood. At the same time, he had pain sensations, severe somatizations, and concentration problems as well as major deficits in physical performance and skills. Nevertheless, observers documented some expression of positive emotions (e.g., joy, pride) and behavior (e.g., smiling, laughing) during the exercise and sport sessions, for example, “Once in a while he even showed [positive] emotions and he smiled when he scored a basket or caught a ball.” As the program progressed, positive affective states were noted more frequently and as a big change by all observers. The trauma-expert was amazed at the positive change and explained that it was rare to see a patient used to so much agony laughing wholeheartedly. Rashid himself explained in the final interview that he had only felt happy 2 days a week, either when he came to therapy or to the sport program. In Rashid’s case, experiencing pleasure and enjoyment seems even more meaningful taking into account the loss of his family, his refugee background, and his mental health problems. Thus, experiencing joy and happiness may have had—at least momentarily—a vitalizing and antidepressant power, a restorative effect (cf. Caddick & Smith, 2014) with regard to his living with PTSD, depression, and his suicide attempts.

It is worth noting that “he only started to enjoy himself during the game and even laughed”; the positive affective states were more frequent in playing situations. Particularly, playing ball games seemed to get him out of his depressive moods and recurrent negative thoughts. Furthermore, positive changes in affective states from the beginning to the end of a session were often observed.

##### Group experiences and a sense of belonging

According to his therapist, Rashid could not establish any solid relationship or tangible friendship in the sport and exercise group, and also the colleagues with whom he was later going to the fitness club “were not directly friends, it was more like being in the same boat.” Yet, Rashid seemed to enjoy the regular contact with the other participants. He even emphasized the sharing of positive emotions as something he most liked about the program: “Laughing, laughing together with others cheerfully.” His therapist highlighted, “his joy when the people came to meet each other; this was something really astonishing.”

At the beginning of the program, Rashid had difficulties engaging with the trainers and the other participants. He trusted his therapist, to whom he once said “you are the only one I know here [in Austria] and I do not know [any other] good persons.” Rashid was for a long time introverted and would not look at anybody. The interaction within the group was determined by doing exercise and sport together, but no further communication took place. In addition, communication with some participants was hindered by them speaking different languages. Yet, the simple fact that the practice of exercise and sport was taking place in a group environment and not individually was much valued by Rashid: “[What was special about the programme?] The group, being together, that was good . . . . Here you are accepted, you are together. If you are strong or weak, that doesn’t matter.” Furthermore, he also got positive feedback from others on several occasions, which helped his sense of belonging, thereby tackling feelings of loneliness and isolation and establishing confidence (cf. Assis et al., 2008).

Toward the middle and end of the program, Rashid did show more of an attempt to make contact with people other than his therapist. For the first time, he established a relationship with the trainers. He also began to speak about more personal issues, such as his sleeping disorders and the many medical drugs he had to take, his suicide attempts, or to show his self-inflicted wounds. At this time, his interactions with other participants of the program increased. In the final interview, Rashid said,

[The] people were from Chechnya, Iran . . . that was good. . . . It was very good, I spoke with people. . . . I like the group, they speak to me. In the park I don’t speak to anybody. Here in the group it is good.

This may seem a small step, but its meaningfulness is becoming more visible, when we consider that Rashid had missed out on stable relationships since his childhood and had an attachment disorder. His therapist explained that “stability in a relationship is crucial for the client, even today.”

##### Experiences of mastery, accomplishments, and perception of self-efficacy

Although Rashid showed major motor-coordinative limitations in the physical training exercises, particularly at the beginning of the program, he surprised the observers with quick and coordinated movements and skillful task performance in the basketball game and other ball games. His successful task performance was more often observed during playing. During playing, his attentional focus was more on the present task performance, facilitating mastery experiences and accomplishments.

Throughout the program, Rashid had many experiences of successful performance, even in spite of severe pain sensations. He managed to perform the exercises until the end despite his shin pain, perceiving his coping competences; he also experienced how he can use exercise to cope with tension and pain (see Coping With Pain).

Rashid’s competences and accomplishments were positively valued by himself, by the trainers as well as by the other participants, for example, he “rejoiced about a point”; he even “showed some pride” and “enjoyed the moment of success” in the group; “participants applauded him,” “he received recognition,” and “positive feedback.” These moments made him visibly happy. These experiences of mastery and accomplishment may have affected on his perception of self-efficacy, as he became more aware of his own competences and improvements in skill (cf. Benight & Bandura, 2004; Samson & Solmon, 2011). The received feedback, encouragement from within the group, and positive affective experiences may have endorsed his self-efficacy beliefs as well (cf. Samson & Solmon, 2011). His mastery attempts may also have served to increase his enjoyment of the activity, thereby having a motivational affect (cf. Motta, McWilliams, Schwartz, & Cavera, 2012; Samson & Solmon, 2011). Experiencing mastery and accomplishments may have positively influenced his activation, perseverance in performing, volition to resist fatigue, and hence finally his performance again (cf. Samson & Solmon, 2011; Teixeira, Carraça, Markland, Silva, & Ryan, 2012). Thus, his experiences of mastery seemed to play a central role and to relate to various other themes and effects (see Figure 1).

##### Activation and motivation

At the beginning of the program, the observers did not have the impression “that he wants to be here and that this is something he likes.” However, his attitude toward the sport and exercise program and his motivation for participation seemed to change over time, leading him to participate more and more actively and with greater perseverance, probably facilitated through his mastery and positive affective experiences (see previous themes). He was sometimes able to motivate himself to participate even when he was in pain. Furthermore, he actively participated in shaping some sessions by suggesting different exercises. His activation and motivation seemed particularly high while playing basketball or other ball games. The way he played was observed as “extremely committed and active.” Furthermore, his motivation for exercising in general increased. He had started to engage in exercise outside the program by running, which showed an increase in his autonomous motivation. For some observers, it was surprising to hear that even after participation in the program, Rashid continued to exercise autonomously. According to the therapist, Rashid bought membership to a fitness club where he trained 3 times a week with colleagues or alone. “There he runs on a treadmill. In the first months he predominantly did aerobic training, and now he has even started slowly with anaerobic things, because he thinks ‘I need muscles,’ and it completely works out for him.” The fact that, 1 year after the program, Rashid is succeeding in exercising on his own several times a week is notable. This finding suggests that he is coping with some effectiveness with major challenges relating to his depression disorder (e.g., low vitality and energy levels), his traumatic experiences (e.g., somatized pain), and his postmigration situation (e.g., isolation). Moreover, a more active living was observed after the program, pointing toward higher energy levels and a more generalized *motivation for living* (cf. Caddick & Smith, 2014, p. 15), enjoying his present life a little bit more: “He goes to the Danube and there he goes for walks on Danube Island, because ‘it is so nice.’ And he also feels more able to handle his life here.”

#### Distraction effect

The analysis of the following themes suggest that during playing Rashid began to pay full attention to present task performance, which along with experiences of mastery and being present in the *here and now*, pointed toward a distraction effect. Notably, although engaged in playing, Rashid was able to tune out negative thoughts, pain sensations, intrusive memories, and flashbacks from the past, providing a respite from PTSD symptoms.

##### Being present, in the here and now

Particularly at the beginning of the program, Rashid seemed absent-minded and his attention and thoughts drifted away quickly. His therapist explained that Rashid “had permanent intrusions—which are flashbacks from past experiences—that you cannot stop and then you do not know where you are, and therefore, he was like in a trance.” However, in the final interview, Rashid stated that “in the sport I am here, and later I am back in my village,” expressing that during sport he is in the present, and afterwards, he is again in the past, remembering experiences from the village he was living in, suffering recurrent thoughts and unwanted intrusive memories about the past, reexperiencing traumatic events. It seems that practicing sport was a *respite* (cf. Caddick et al., 2015) from recurring thoughts of the past, intrusive memories, and the reexperiencing of traumatic events, a moment in which he tangibly experienced the present and positive affective states. However, Rashid also expressed that “after the sport I again have a lot of thoughts. For one hour of sport I am happy, and then? In the metro, in the house, what shall I do?” Nevertheless, the therapist noted that Rashid is now more often in the present, in the *here and now*; he is more clear in his mind and less in a trance; and he expresses spontaneous emotions, such as showing happiness about a meeting or being in nature.

##### Attentional focus on present task performance

At the beginning of the program, Rashid showed a limited range of motion and major coordination problems, especially observable in the mobilization and strength exercises which he hardly could perform, and in some he barely participated. Yet, he also surprised with quick and coordinated movements in the ball games: “He showed incredible skills in the ball game, compared to his rather modest skills in the other activities of the session.” Surprisingly, his skills were valued as better in more complex situations, that is, in play situations in ball games where more attention, quick reactions, and the coordination of several movements were demanded. Conversely, in the physical exercises, his attentional focus seemed not to be on exercising, he was perceived rather as absent-minded, distracted, confused, and abstracted, and he showed few emotional reactions to present stimuli. One observer wrote, “I had the impression that he had difficulties in doing the [physical] exercises deliberately.” That may mean that the high physical skills demonstrated during playing may have been performed in some way automatically, without paying deliberate attention to his body, to somatic arousals, his own physical movements, or limitations. His attentional focus during playing seemed to be completely on the present task performance. He seemed to be more engaged and present while playing, reacting to present stimuli. Pain sensations and negative thoughts seemed not to disturb the present experience, indicating a distraction effect. The attentional focus on present task performance during playing seemed to facilitate fewer physical restraints for Rashid, better movement performance, and more mastery experiences. Furthermore, a higher degree of activation and motivation as well as more positive affective states were observed concurrently while playing. Thus, the attentional focus on present task performance stimulated several underlying processes of the distraction effect and the motivational-restorative effect.

#### Exposure effect

The findings from the following themes suggest that the attentional focus on bodily sensations during physical exercise exposed Rashid to negative body- and self-experiences; at the same time, positive experiences of own coping with pain, knowledge acquisition, and a reassessment of the negative association between pain and exercising may have facilitated improvements in body- and self-awareness, affect regulation, and the relationship with his body and exercising.

##### Body- and self-experiences

At the beginning of the program, Rashid was not able to identify which muscles or parts of the body he was feeling in a certain mobilization or strength exercise. “To feel the body is alien to him and he does not really achieve it.” The therapist observed that “when the programme began, he did not know where his pulse was.” The data suggested that he slowly increased his body awareness. When asked about the perceived changes through the program, his therapist answered, “Improvements of body awareness.” For example, Rashid could, by this time, point toward the area of the body he felt during a mobilization or strength exercise. He had got to know his body better.

At the same time, although body- and self-awareness seemed to increase, he became aware of negative sensations (e.g., pain, perceived limitations). He told his therapist that he felt as if “he is an old man in a young body” and according to the therapist,

he became more aware about this. Somehow his self-image was affected somewhat by seeing himself not performing in comparison to the others. This caused some insecurity and affected his self-image, which then disappeared, I would say; playing basketball it definitely disappeared. There he laughed.

Thus, the attentional focus toward himself and his body may have triggered negative sensations.

Nevertheless, the findings suggest that sport and exercise also facilitated experiences that gave him the opportunity to learn more about his own body, to reassess his bodily sensations, and to experience his body and, thus, himself as a whole in a more positive way (cf. Harris, 2007; Levine & Land, 2016). For example, he became aware that physical effort is not necessarily related to something negative or threatening. According to the therapist,

Rashid can now manage his symptoms in a better way, he understands that: “when my heart beats and I perceive this, it does not mean that I have to fall down dead just because I feel my heart.” . . . He noticed that: “if I am physically active, then I relax and calm my nerves. I am physically tired, but afterwards I am more balanced, I have less stress in me, I am less anxious and things agitate me less.”

Thus, the program “was also psychoeducational for him,” and the acquisition of knowledge and positive body- and self-experiences were important for his affect regulation (cf. Van der Kolk et al., 2014), reduction in anxiety sensitivity (cf. Asmundson et al., 2013), and for reestablishing a more healthy relationship with sport and exercise as well as with his own body and self (cf. Levine & Land, 2016).

##### Coping with pain sensations

Throughout the program, in nearly every session, Rashid not only complained about pain, especially in the shin, but also headaches and feelings of dizziness. Rashid told his therapist,

It is impossible for me to run; I always have pain in my shin. That is from the past, from the people who murdered my parents, they beat me so hard with an assault rifle that I could not move for days, and since then I have had this pain.

Pain sensations and associated psychological distress made him sometimes stop exercising. Furthermore, the findings suggest that a negative association between exercise and pain was prevalent in the first half of the program. Although the sensations of pain persisted throughout the program, the association between exercising and pain seemed to change and a better coping ability may have been achieved. The therapist noted that “this pain remained until the end, but today I know that he goes running three times a week!” Furthermore, Rashid’s comments suggested that practicing sport and exercise had some positive impact on his pain sensations, which often decreased toward the end of a session.

He also learnt that he can perform the exercises in spite of the pain and have success in sport, and that he can even enjoy exercising. In the final interview, Rashid stated that he had acquired knowledge in the sport and exercise program about how to handle tensions and pain: “I do these exercises [e.g., arm circling] and then my tension improves, then [my] headache is better. . . . Before I did not understand what to do when [I have] pain. Now, I know it.” Furthermore, he explained that he was using these “exercises for headache at home.” It is possible that the negative association between exercising and pain was transformed through the experience of positively coping with pain sensations within a session, which seemed important for creating a healthy relationship with sport and exercise, as well as with his body (cf. Levine & Land, 2016). Although the shin pain sensations persisted throughout the program, the pain sensations hardly stopped him from running or performing exercises in the second half of the program. Despite the pain sensations, Rashid managed to improve his commitment to exercising and even adhered to regular exercise after the program had stopped. Thus, the improvements in body awareness, knowledge acquisition, and the perception of positive bodily experiences seemed important aspects with regard to Rashid’s dealing with pain sensations and somatic arousals (see Figure 1).

## Discussion

The findings of this study extend the previous literature by presenting a case study which described a holistic set of diverse processes and effects that can take place in sport and exercise therapy with war and torture survivors. The encountered complexity of interactions between various themes endorses the assumption that one process or mechanism cannot explain the effects of sport and exercise on mental health (Faulkner et al., 2015; Fetzner & Asmundson, 2015).

The findings suggest the occurrence of two different attentional foci: one on bodily sensations and one on present task performance during playing (see Figure 1). The attentional focus on bodily sensations, which occurred in this study mainly during physical exercises, may have facilitated an exposure effect. The participant was getting more aware of his body and self; at the same time, he was also exposed to negative sensations. These negative sensations sometimes stopped him from exercising and constrained his physical performance during the physical exercise. Exposure to negative sensations may make exercise less enjoyable and more distressing, and may “encourage people to stop rather than to continue exercising and, consequently, quash the therapeutic opportunity” (Fetzner & Asmundson, 2015, p. 310). This may particularly happen if participants are not accompanied by a therapist and the negative sensations are not cared about. This was not, however, the case with Rashid.

Rashid augmented his body awareness and reassessed his negative associations between exercising and pain. Pain was frequently observed throughout the program, but his coping behavior and awareness of the benefits of sport and exercise seemed to improve. Moreover, the findings suggest that he even became more aware that bodily sensations (e.g., pain or fast heart beats) are not necessarily catastrophic (e.g., relate to a traumatic event), and that these bodily sensations are somehow natural when practicing sport and exercising. This finding aligns with several studies that have shown that exercise-based (interoceptive) exposure to feared bodily sensations (e.g., fast heart rate, sweating, shortness of breath, muscle tensions) may provide anxiolytic effects by demonstrating the nonthreatening nature of these bodily sensations and reducing anxiety sensitivity (Asmundson et al., 2013). In addition, Rashid’s experiences of mastery seemed to help him cope with pain sensations and may have increased his coping self-efficacy beliefs, that is, the sense of competence in coping with threats, for example, feared bodily sensations, and reduce worries, fears, and anxiety (Benight & Bandura, 2004). “Mastery stemming from exercise-related successes appear to counteract catastrophic thinking characteristic of anxious individuals, further increasing feelings of mastery and self-efficacy” (Asmundson et al., 2013, p. 368). These findings also align with the results of the RCT on trauma-sensitive yoga by Van der Kolk et al. (2014), who concluded that “if traumatized individuals can learn to identify and tolerate physical sensations, they are likely to increase emotional awareness and affect tolerance” (p. e564).

The attentional focus on playing seemed to facilitate two different effects, that is, a motivational-restorative and a distraction effect. On one hand, the attentional focus on task performance during playing facilitated mastery experiences and concentration on the present *here and now*, achieving a distraction from negative (illness- and migration-related) thoughts and pain sensations. Remarkably, the participant stated that during sport he is *here*—present and happy—and after the sport he is back in his village with his trauma-associated thoughts and intrusive memories. It seemed that practicing sport and exercise was a therapeutically meaningful moment of recovery (cf. Harris, 2007), a respite from recurring thoughts of the past, intrusive memories, and the reexperiencing of traumatic events. Caddick et al. (2015) described similar processes, analyzing how combat veterans experienced a respite from PTSD symptoms through lived experiences while surfing. In the present study, Rashid had tangible and vivid experiences during sport and exercise, experiencing his body, being in motion, his skills, his accomplishments, and affective states in the present moment, and he became more aware of present processes regarding himself. These experiences were directly livable during sport and exercise; they were also talked about briefly in the same session. The lived experiences also became topics in the psychotherapy sessions. This showed possible synergistic effects between the therapies, and the benefits of using sport and exercise as an adjunctive therapy connected to a holistic therapeutic approach. This connection was ideal in the participant’s case, as the psychotherapist was taking part in the sport and exercise program and the communication channel between the psychotherapists and the sport and exercise therapist was direct and constant.

On the other hand, the attentional focus on playing facilitated experiences of positive affective states, mastery, activation, and a sense of belonging, providing a motivational-restorative effect on Rashid regarding his living with PTSD and depression, and regarding his adherence to exercise. Experiencing positive affective states is therapeutic, for example, “feeling good during and/or after physical activity is motivational, serves as an important health outcome in itself, and contributes to quality of life” (Faulkner et al., 2015, p. 212). Furthermore, the description of Rashid being completely focused on the present task performance during playing, performing it somehow automatically, achieving the experience of mastery and enjoyment is reminiscent of a flow-state (Csikszentmihalyi, 2014). Flow experiences are said to have important health, distraction, stress-releasing, and motivational effects (Ley et al., 2017). With regard to the here proposed processes, the flow concept seems to englobe the distraction and the motivational-restorative effects (cf. Ley et al., 2017).

Furthermore, the findings regarding the motivational-restorative effect align to the affect circumplex model and core affect hypothesis (Ekkekakis, 2008; Rebar, Faulkner, & Stanton, 2015), which conceptualized affective states by integrating the dimensions of affective valence and activation. The sport and exercise program increased Rashid’s activation and enjoyment, that is, the pleasant-activated core affect, which opposes the unpleasant-deactivated core affect typical in depressive disorders (Rebar et al., 2015). Thus, the case study illustrates some processes that may help to enhance this core affect. However, whether the program also increased the pleasant-deactivated core affect (e.g., satisfaction, calmness, relaxation; Rebar et al., 2015), which opposes PTSD symptoms like hyperarousal and anxiety, seems unclear in this study. Yet, we discussed above potential anxiolytic effects in relation to experiencing the nonthreatening nature of somatic arousals in sport and exercise and coping self-efficacy, which may relate to a certain degree of deactivation (e.g., being less stressed, more balanced and relaxed) and reduction of anxiety sensitivity and hyperarousal.

Thus, both attentional foci may provide important therapeutic opportunities (cf. Fetzner & Asmundson, 2015). However, facilitating and directing attentional focus in either direction (away or toward bodily sensations) should be used intentionally, in an appropriate environment and moment. Directing attentional focus away from the bodily sensations—to provide distraction and pleasure instead of exposure—can also be seen as a strategy for avoiding negative sensations, particularly those relating to traumatic events; thus, a defense mechanism “to protect their traumatized bodies” (Levine & Land, 2016, p. 9). Rashid seemed more receptive to feeling and accepting bodily sensations toward the second half of the program; meanwhile, distraction through playing was possible from the very beginning.

The findings suggest that Rashid increased his motivation for and autonomy in exercising, which in turn may have stimulated a more active living and motivation for living. In contrast to previous studies with veterans with combat trauma (Caddick & Smith, 2014), the findings of this study suggest that improvements in autonomy were visible and meaningful for Rashid at quite an early stage of recovery. The program *Movi Kune* seemed to be autonomy-supportive (cf. Ryan & Deci, 2000), for example, by means of providing knowledge, opportunities for self-direction, and choices (e.g., to opt-out or opt-in during a session, to self-regulate intensity) as well as participation in shaping the session (e.g., proposing exercises or deciding on which sport to play). Autonomy is one important basic psychological need in the self-determination theory (SDT) and related to intrinsic motivation and well-being (Ryan & Deci, 2000; Teixeira et al., 2012). Autonomous motivation is crucial for adopting and maintaining physical activity in people with mental illness (Vancampfort et al., 2015). Another important basic psychological need in SDT is the sense of competence, the need to be effective in dealing with the environment (Ryan & Deci, 2000). Rashid’s experiences of mastery and accomplishments—together with positive self and group experiences (i.e., becoming aware of his own skills and coping abilities as well as feedback and encouragement from the trainers and other participants)—relate to self-efficacy beliefs and sense of competence (cf. Benight & Bandura, 2004; Samson & Solmon, 2011). In this case study, the mastery experiences played a central role for all three effects (see Figure 1).

The third basic psychological need in SDT is relatedness, which refers to the need to feel connected to others, to attain a sense of belonging, and to establish close personal relationships (Ryan & Deci, 2000; Teixeira et al., 2012). The findings of this case study suggest that the sport and exercise program offered Rashid valuable opportunities for joyful social interaction, positive feedback from others, and a sense of belonging. Rashid highlighted the group experiences as being very important; similar to the combat veterans in the study of Caddick et al. (2015), who related the positive social interaction during surfing to feelings of safety and respite from problems associated with PTSD, enabling “them to relax and enjoy the activity of surfing” (p. 82). Levine and Land (2016), however, indicated additionally that “with many victims of trauma, building a healthy physical relationship, both with oneself and with others, is difficult” (p. 10). This may have been the case with Rashid as well: The relationships Rashid constructed in the program did not reach solidity or include a real friendship. Maybe, the program did not provide the necessary time and communication channels to construct stable relationships. Nevertheless, practicing sport and exercise in a safe and trustworthy group environment provided Rashid with the opportunity to get out of his isolation and to orient himself toward the outside, socializing with others, which is a cautious and meaningful step toward constructing relationships and relatedness. Thus, the program should be connected with long-lasting opportunities for sport and exercise, for example, in sport clubs or fitness centers, which may provide opportunities for further social interaction and inclusion, and thus for the construction of stable relationships and relatedness.

The findings of this study point toward some important caution and challenges for sport and exercise trainers/facilitators. Participants may be exposed in and through sport and exercise to negative sensations and to triggers of distress, intrusive memories, or even retraumatization. For example, Rashid’s persistent shin pain sensations were associated with memories from the past when his parents were murdered in front of him and he was beaten with an assault rifle. Pain sensations and associated psychological distress made him sometimes stop exercising. Furthermore, Rashid also became aware of his own limitations by seeing himself as not performing in comparison with the others, which may have negatively affected his self-concept. Dealing with these challenges in a constructive manner seems paramount for trauma-sensitive sport and exercise programs (Ley et al., in press).

### Limitations

This case study approach comes with limitations. This begins with an awareness that the case description is partial and incomplete, due to the constraints of the data production methods (i.e., interview and observation); the inability of the participant, therapist, and observers to capture, recall, and narrate completely the happenings and experiences; and the biases of the researchers. For example, the verbal and nonverbal expressions of the participant during the sport and exercise sessions were captured by the observers, providing an important additional account of the participant’s experiences; however, observers’ own perceptions, experiences, and interpretations may have had an influence in the writing of the corresponding field notes.

The aim of this study was to provide an in-depth understanding of a single participant. Therefore, no generalization from a positivist perspective was envisaged (see *Methodology*); nevertheless, transferability of findings of this study to similar cases and naturalistic generalizations may be considered by the user (Day & Wadey, 2016; Sparkes & Smith, 2014; Stake, 1995). We consider that the theoretical concepts built from the data were strongly relating to existing theories and concepts, that is, self-efficacy and SDT, the core affect, respite and flow concepts as well as to antidepressive and anxiolytic effects. We suggest that the proposed processes and effects may be relevant in similar sport and exercise programs with people with similar mental health problems, that is, PTSD, anxiety, and depression. However, it is important to acknowledge that there are alternative and diverse experiences in sport and exercise, and that the processes and effects may differ among the participants, for example, due to the heterogeneity of the participants, group dynamics, and recovery processes. Furthermore, the sport and exercise was offered as an adjunctive therapy. The participant also regularly attended psychotherapy, received medication, and psychiatric intervention. It is assumed, and in fact—for the benefit of the participant—desired for synergistic effects to occur between the therapies. Every effort was made to refer to data that related more inherently to sport and exercise processes and effects.

## Conclusion

The findings of this case study describe diverse effects—that is, motivational-restorative, distraction, and exposure effects—of a sport and exercise therapy program on a war and torture survivor, showing the complexity of interactions between various processes and effects. The effects seemed to contribute to an improved sense of well-being of the participant (i.e., enjoying life more, having less depressive moods, being more active and motivated for living), a respite from PTSD symptoms, more presence, body- and self-awareness, and exercise adherence. Thus, the findings support the application of sport and exercise in the rehabilitation and recovery process of war and torture survivors and people living with PTSD, adding some holistic insights to the existing literature and implications for practitioners about how sport and exercise can be implemented, which precautions need to be considered, and which effects and processes may be targeted.
